# Adipose‐derived and bone marrow aspirate concentrate injections for osteoarthritis: A scoping review

**DOI:** 10.1002/pmrj.70147

**Published:** 2026-05-02

**Authors:** Leonardo P. Oliveira, Aaron Lear, Joseph Elphingstone, Derek Stokes, Rachel Chamberlain, Timothy Tiu, David Price, Megan Agnew, Stephanie Kliethermes, Sarah Sund, Leslie A. Christensen, Shane Shapiro, Kenneth Mautner

**Affiliations:** ^1^ Cleveland Clinic Florida Weston Florida USA; ^2^ Cleveland Clinic Akron General Akron Ohio USA; ^3^ University of Alabama at Birmingham Birmingham Alabama USA; ^4^ University of Colorado Boulder Colorado USA; ^5^ University of New Mexico Albuquerque New Mexico USA; ^6^ University of Miami Miami Florida USA; ^7^ Atrium Health Charlotte North Carolina USA; ^8^ University of Wisconsin Madison Wisconsin USA; ^9^ Ebling Library for the Health Sciences University of Wisconsin School of Medicine and Public Health Madison Wisconsin USA; ^10^ Mayo Clinic Jacksonville Florida USA; ^11^ Emory University Atlanta Georgia USA

## Abstract

**Background:**

Osteoarthritis is a leading cause of disability worldwide, and cell‐based treatments including adipose‐derived and bone marrow aspirate have been sought by the lay and medical community as treatment options.

**Objective:**

To perform a scoping review of published literature on cell‐based injections allowed by the U.S. Food and Drug Administration for the treatment of osteoarthritis to synthesize existing evidence and identify research gaps for future evaluation.

**Methods:**

A comprehensive search of five databases was executed from inception through January 2, 2025. Studies that met the inclusion criteria were original research studies written in English on Food and Drug Administration‐allowed cell‐based treatments in adults with osteoarthritis of any joint.

**Results:**

The database search yielded 4257 unique records. After screening, 84 studies met the inclusion criteria, encompassing 9996 patients and a total of 10,508 procedures. The primary research study designs were cohort studies (*n* = 62), focused on treatment of knee osteoarthritis (*n* = 63), and described bone marrow aspirate (*n* = 42) and adipose‐derived (*n* = 42) treatments. Postprocedure monitoring ranged from 6 weeks to 5 years, with most studies ≤1 year (*n* = 59). Patient‐reported outcomes were reported in 83 of 84 studies; few provided imaging outcomes including magnetic resonance imaging (*n* = 9) or radiographs (*n* = 2).

**Conclusion:**

This review identified limited randomized controlled trials, limited studies outside the knee, limited description of cell‐based treatments, and treatment protocols, along with inconsistent patient‐reported outcomes limited to 1 year in most studies. We propose establishing reporting guidelines in research on cell‐based therapies.

## INTRODUCTION

Osteoarthritis (OA) is a leading cause of disability, affecting 595 million individuals worldwide and primarily localized to the knee.^1^ Multiple risk factors for OA have been described, including advanced age, obesity, rheumatologic conditions, and trauma to joints. A variety of nonsurgical treatments are recommended by professional societies including physical therapy, weight loss, bracing, oral nonsteroidal anti‐inflammatory medications (NSAIDs), and injectables such as corticosteroids and hyaluronic acid for the knee.^2^, ^3^, ^4^, ^5^ Orthobiologics are biologic substances intended to treat musculoskeletal disease and are considered for nonsurgical management. Recognizing the lack of consensus among health care professionals,^4^ improved outcomes have been reported using platelet‐rich plasma (PRP) with higher platelet counts^6^, ^7^ and in multiple meta‐analyses describing primarily management of OA.^8^, ^9^, ^10^

However, orthobiologics often are not covered by insurance and the resulting out‐of‐pocket costs may limit access. Furthermore, underrepresentation of non‐White patients in outcomes studies, including in knee OA, may limit the generalizability of findings.^11^ In addition to the need for inclusion of more diverse patient populations, experts across multiple societies, including the American Academy of Physical Medicine & Rehabilitation, have identified additional aspects of PRP delivery that warrant standardization (cross‐reference). Joint arthroplasty surgery for large joints such as the knee, hip, and shoulder remains the primary surgical treatment for OA,^5^ but 5 million individuals are estimated to be in the orthopedic treatment gap in which nonoperative treatments have failed and the patient is unwilling or unable to undergo surgery.^12^ Despite arthroplasty's high survivorship,^13^ it does not always predictably restore whole body function,^14^ with up to 20% of patients regretting knee surgery^15^ and some developing infection.^16^

While the science and evidence supporting orthobiologic treatments continue to evolve, PRP and cell‐based injections (CBI) are increasingly being explored as treatment options for OA. The goal of these interventions is to provide a minimally invasive alternative to surgery. Some studies suggest PRP may be effective in relieving pain associated with knee OA.^10^, ^17^ CBI such as bone marrow aspirate concentrate (BMAC) and microfragmented adipose tissue (MFAT) are also being explored, with proposed mechanisms centering on the paracrine effects of mesenchymal stem cells (MSCs) contained in the injectate.^18^, ^19^ These in vivo secretory functions affecting tissue function, repair, and inflammation were the reason why one of the pioneer scientists in this field, Dr. Arnold Caplan, recommended renaming these cells as medicinal signaling cells.^20^ The growing interest in these treatments has generated important yet unanswered questions regarding optimal harvesting techniques, dosing, delivery, and underlying mechanisms of action.^21^ Treatment protocols require both pre‐ and postinjection recommendations. Additionally, there is no optimal standard with varying methodologies ranging from allogenic and autologous to culture‐expanded MSC injections.^22^, ^23^, ^24^

The purpose of this scoping review is to synthesize the existing literature for U.S. Food and Drug Administration (FDA)‐allowed CBI for the management of OA and identify knowledge gaps. The current FDA guidance document specifies that the tissue harvested can undergo only minimal manipulation (cannot be culture expanded or enzymatically modified) and should follow homologous use.^25^ The outcomes of this review will be highlighted as part of the American Medical Society for Sports Medicine Collaborative Research Network Research Summit, Bridging the Gaps: Orthobiologics in Sports Medicine, in Seattle in April 2026.

## METHODS

The scoping review was developed and conducted in accordance with the 2021 Joanna Briggs Institute methodology for scoping reviews and Arksey and O'Malley methodological framework for scoping reviews with recommendations by Levac et al. Results are reported using the Preferred Reporting Items for Systematic Reviews and Meta‐Analyses Extension for Scoping Reviews.^26^, ^27^ A scoping review protocol was developed by the authors and registered on the Open Space Framework website before the start of title and abstract screening^.^ A writing group was formed via an open call to physician members of the American Medical Society for Sports Medicine. A web‐based collaboration software platform, Covidence (Veritas Health Innovation, Melbourne, Australia), was used to streamline the scoping review process.

### Search strategy

The search strategy focused on two general concepts:(1) CBI currently performed in the United States and (2) OA. Using these concepts, the review team worked with a research librarian (L.A.C.) to develop and execute a comprehensive literature search using controlled vocabulary and title/abstract terms associated with the two general concepts. The search strategy was developed in PubMed, then translated into the following databases: MEDLINE (EBSCO), Embase.com (Elsevier), Cochrane Library (Wiley), and Science Citation Index‐Expanded and Emerging Sources Citation Index as a multi‐file search of the Web of Science Core Collection (Clarivate). All searches were conducted from database inception through January 2, 2025. An exclusion filter was used to remove animal studies prior to screening. In PubMed, results were limited to publisher submission and in‐process citations. No other filters (publication type, date, language) were applied to the results. Full search strategies are available in the published protocol on Open Space Framework (https://osf.io/k2qrv/files/tjsab).

Results were downloaded to a citation management software (EndNote version 20.6) and underwent manual deduplication  (L.A.C.) using the method described by Bramer. Unique records were uploaded to Covidence systematic review software (Veritas Health Innovation, Melbourne, Australia) for review by team members. In addition to the initial database searches, five systematic reviews included in the final review were selected by (K.M.) for supplemental handsearching of references for additional studies to include in the review.

### Inclusion/exclusion criteria

Included studies met the following criteria: adult participants (18 years of age and older), diagnosis of OA, CBI allowed by the FDA (autologous cellular therapies, BMAC, bone marrow‐derived stem cells, mesenchymal stem/stromal cells, and adipose products),^25^ human studies, injectable treatments, and with full text available in English. Studies were excluded if they were studies of children or adolescents; diagnoses of rheumatoid arthritis, psoriatic arthritis, inflammatory arthritis, or gout; cellular‐based injections not allowed by the FDA (culture expansion of cells, adipose stromal vascular fraction [SVF], birth tissue products including umbilical cord products or Wharton's jelly, and allogenic cellular therapies); animal or in vitro studies; had a surgical component; published abstracts or protocol only; or combined a cell‐based therapy with another treatment. All contexts were included in the search, but the focus was on CBI currently permitted for use in the United States.

The scoping review considered the following types of sources: experimental and quasi‐experimental studies, analytical observational studies. Nonsystematic literature reviews, case reports, case series, opinion papers, and editorials were not included. Systematic reviews were obtained to identify any primary references not caught in the initial search; however, they were not included in the synthesis of existing literature.

### Study selection

Records were imported into Covidence for title and abstract screening. Before screening, a meeting was held with the reviewers (A.L., D.S., D.P., J.E., L.O., R.C., T.T.) to review the scoping review process and inclusion criteria, which included a pilot test using 10 studies to ensure the reviewers were consistent in their understanding of the process and criteria. Each title and abstract was screened independently by two reviewers (A.L., D.S., J.E., L.O., R.C., T.T.) in a blinded process. Conflicts were resolved by a third reviewer (K.M., S.S.).

A pilot test was again conducted before starting the full‐text review phase using five studies to ensure the reviewers were consistent in their understanding of the eligibility criteria. The full text of all eligible studies was then assessed against the inclusion/exclusion criteria independently by two reviewers (J.E., D.P., D.S., M.A., L.O., R.C., S.S., S.K.). Any conflicts were resolved by a third reviewer (K.M.).

### Data extraction

A data extraction template was developed (K.M., S.K., and M.A.) in Covidence. Data extractors reviewed a selection of studies to ensure the fields matched the research questions. The data extraction fields consisted of details on the participants, concept, context, study methods, and key findings relevant to the review questions. A meeting was held with the data extractors to ensure everyone understood the process and best practices of data extraction. Two independent reviewers extracted data from the primary research studies, and a third reviewer (K.M.) resolved any conflicts.

### Consultation exercise

Following the recommendations by Arksey and O'Malley, we held a consultation meeting with sports medicine physicians working on ancillary scoping reviews for the research summit. The goal of the meeting was to present preliminary findings and allow for an open discussion.^28^

In preparation for the consultation meeting, summary tables from the data extraction phase were provided to ChatGPT (Open AI, San Francisco, CA) with the following prompt: “Writing a scoping review. Can you analyze the Excel document and help figure out the gaps in platelet‐based therapies for muscle injury.” The AI‐based summaries were then used to provide an initial framework for the study team to further identify, explore, and decide upon important research gaps. All subsequent interpretations, categorizations, and calculations were manually performed by the investigators.

## RESULTS

**FIGURE 1:**
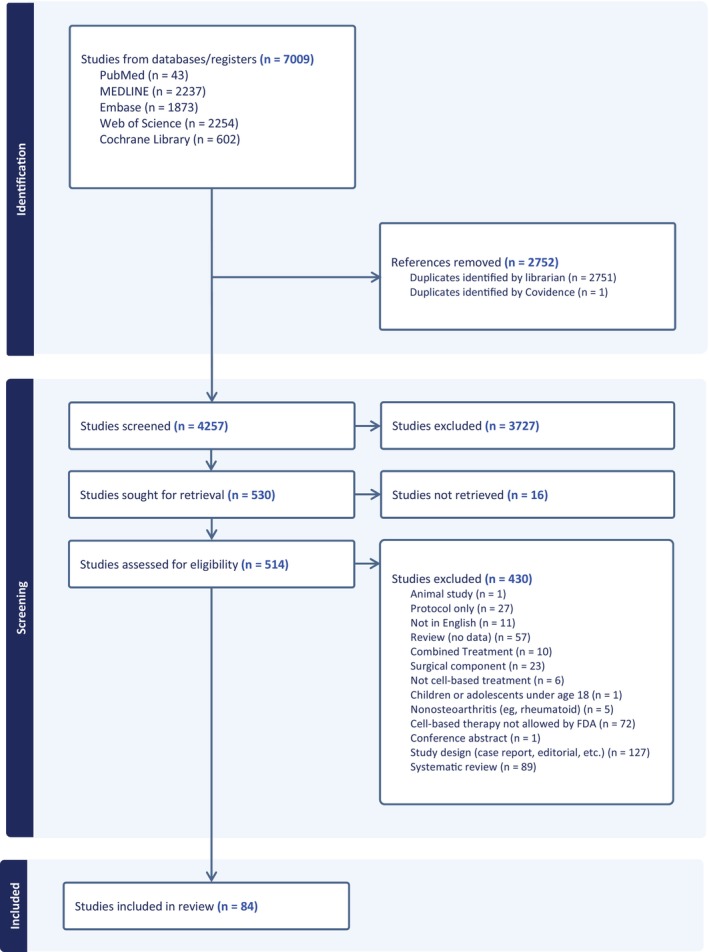
PRISMA flow diagram. FDA, Food and Drug Administration; PRISMA, Preferred Reporting Items for Systematic Reviews and Meta‐Analyses.

The initial database search identified 4257 unique studies for title and abstract screening. After title and abstract screening, 530 studies underwent full text review, resulting in 84 primary research studies that met the inclusion criteria and were used in our scoping review (Figure 1).

Of the 84 primary research articles (Figure 1),^29^, ^30^, ^31^, ^32^, ^33^, ^34^, ^35^, ^36^, ^37^, ^38^, ^39^, ^40^, ^41^, ^42^, ^43^, ^44^, ^45^, ^46^, ^47^, ^48^, ^49^, ^50^, ^51^, ^52^, ^53^, ^54^, ^55^, ^56^, ^57^, ^58^, ^59^, ^60^, ^61^, ^62^, ^63^, ^64^, ^65^, ^66^, ^67^, ^68^, ^69^, ^70^, ^71^, ^72^, ^73^, ^74^, ^75^, ^76^, ^77^, ^78^, ^79^, ^80^, ^81^, ^82^, ^83^, ^84^, ^85^, ^86^, ^87^, ^88^, ^89^, ^90^, ^91^, ^92^, ^93^, ^94^, ^95^, ^96^, ^97^, ^98^, ^99^, ^100^, ^101^, ^102^, ^103^, ^104^, ^105^, ^106^, ^107^, ^108^, ^109^, ^110^ 40% (*n* = 34) were prospective cohort studies, 31% (*n* = 26) case–control or retrospective cohorts, and 23% (*n* = 19) were randomized controlled trials (RCT). There were three nonrandomized clinical trials^41^, ^55^, ^59^ and two registries^44^, ^46^ (Tables 2 and S1). From these studies, 9996 patients were included encompassing 10,508 joint injections.

Of the 19 RCTs, 47% (*n* = 9) involved BMAC as the primary intervention^29^, ^30^, ^51^, ^52^, ^60^, ^80^, ^96^, ^97^, 53% (*n* = 10) adipose‐derived cellular products,^35^, ^36^, ^37^, ^49^, ^76^, ^79^, ^93^, ^107^, ^109^ and 5% (*n* = 1) BMAC+PRP.^47^ The comparator treatments in the RCTs included nine studies using PRP,^29^, ^30^, ^35^, ^36^, ^37^, ^76^, ^79^, ^107^, ^109^ two using saline,^96^, ^97^ two using corticosteroids,^52^, ^93^ one using hyaluronic acid combined with PRP,^49^ one compared against exercise,^47^ and one against corticosteroid, SVF, and umbilical cord treatments^80^ (Table 1).

**TABLE 1:** Included randomized controlled trials.

Author	Year	Joint	CBI	Control	Blinding	Pretreatment protocol	US guidance	Posttreatment protocol	Last assessment ≤12 months	PROMs	Reported adverse events	Reported outcomes relative to control
Baria	2022	Knee	Adipose	PRP	None	Y	Y	Y	Y	KOOS, Tegner, VAS	Y	−/−
Baria	2024	Knee	Adipose	PRP	None	N	Y	Y	Y	KOOS, Tegner, VAS	Y	−/−
Baria	2024	Knee	Adipose	PRP	None	N	N	Y	Y	KOOS	N	−/−
Dallo	2021	Knee	Adipose	PRP + HA	None	N	U	Y	Y	KOOS, MARX, Tegner, VAS	Y	+
Kaszyński	2022	Knee	Adipose	PRP	Single	N	N	N	Y	EQ‐5, KOOS, WOMAC, VAS	N	−/−
Louis	2021	Knee	Adipose	PRP	Double	N	Y	Y	Y	WOMAC, VAS	Y	+
Richter	2024	Knee	Adipose	CSI, saline	Double	N	N	N	Y	KOOS, VAS	Y	+
Winter	2023	Hand/wrist	Adipose	PRP	Single	N	Y	Y	N	NRS, SF‐36, Quick DASH	Y	+
Zaffagnini	2022	Knee	Adipose	PRP	Single	N	Y	Y	N	EQ‐5, KOOS, VAS	Y	−/−
Anz	2020	Knee	BMAC	PRP	None	Y	Y	Y	Y	IKDC, WOMAC	N	−/−
Anz	2022	Knee	BMAC	PRP	None	Y	Y	Y	N	IKDC, WOMAC	N	−/−
Boffa	2022	Knee	BMAC	HA	Double	N	Y	Y	N	KOOS, VAS	Y	+
Dulic	2020	Knee	BMAC	Different injection portals	None	N	Y	N	Y	IKDC, KOOS, VAS	Y	−/−
Dwyer	2021	Shoulder	BMAC	CSI	None	N	Y	Y	Y	EQ‐5, WOOS, VAS	Y	+
Goncars	2017	Knee	BMAC	HA	None	N	N	Y	Y	KOOS, KSS	Y	+
Mautner	2023	Knee	BMAC	SVF, UCT, CSI	Single	N	Y	N	Y	KOOS, VAS	Y	−/−
Shapiro	2017	Knee	BMAC	Saline	Single	N	Y	Y	Y	ICOAP, OARSI, VAS	Y	−/−
Shapiro	2019	Knee	BMAC	Saline	Single	N	Y	Y	Y	ICOAP, VAS	Y	−/−
Centeno	2018	Knee	BMAC + PRP	Exercise	None	Y	Y	Y	N	KSS	Y	+

Abbreviations: +, intervention superior to comparison group; −/−, no difference between groups; BMAC, bone marrow aspirate concentrate; CBI, cellular based injection; CSI, corticosteroid injection; EQ‐5, EuroQuol 5 dimensions; ICOAP, intermittent and constant osteoarthritis pain; IKDC, International Knee Documentation Committee; KOOS, Knee injury and Osteoarthritis Outcome Score; KSS, Knee Society Score; MARX, Marx Knee Measure; N, No; NRS, Numeric Rating Scale; PRP, platelet‐rich plasma; OARSI, Osteoarthritis Research Society International; PROM, patient‐reported outcome measure; Quick DASH, Quick Disabilities of the Arm, Shoulder and Hand Questionnaire; SF‐36, Short‐Form‐36; SVF, Stromal Vascular Fraction; Tegner, Tegner Activity Scale; U, unknown; UCT, umbilical cord stem cells; US, ultrasound; VAS, Visual Analogue Scale; WOMAC, Western Ontario and McMaster Universities Osteoarthritis Index; WOOS, Western Ontario Osteoarthritis of the Shoulder Index; Y, Yes.

Of the studies, 75% (*n* = 63) focused on the knee joint,^29^, ^30^, ^31^, ^32^, ^33^, ^34^, ^35^, ^36^, ^37^, ^38^, ^39^, ^40^, ^41^, ^42^, ^43^, ^44^, ^45^, ^46^, ^47^, ^49^, ^51^, ^53^, ^55^, ^56^, ^57^, ^58^, ^59^, ^60^, ^61^, ^64^, ^65^, ^67^, ^70^, ^72^, ^74^, ^75^, ^76^, ^77^, ^78^, ^79^, ^80^, ^81^, ^83^, ^84^, ^85^, ^89^, ^90^, ^91^, ^92^, ^93^, ^94^, ^95^, ^96^, ^97^, ^98^, ^99^, ^100^, ^101^, ^103^, ^105^, ^106^, ^108^, ^109^, ^110^, ^111^, ^112^ 9% (*n* = 8) on wrist and hand,^46^, ^50^, ^62^, ^63^, ^68^, ^69^, ^82^, ^107^ 5% (*n* = 4) hip,^42^, ^46^, ^88^, ^102^ and 7% (*n* = 6) shoulder^46^, ^48^, ^52^, ^56^, ^86^, ^104^ (Tables S1 and 2). Study locations included 59% in Europe (*n* = 50), 28% (*n* = 24) in the United States, and 6% (*n* = 5) in India. Regarding funding, 68% (*n* = 58) reported no funding, 18% (*n* = 15) private funding, 11% (*n* = 9) federal funding, and 3% (*n* = 3) were funded but no source was reported.

**TABLE 2:** Study characteristics summary.

Characteristic		Adipose (%)	BMAC (%)
Study design	Prospective cohort	20 (47.6)	16 (38.1)
	Retrospective cohort	13 (31.0)	13 (31.0)
	RCT	9 (21.4)	11 (26.2)
	Registry	0	2 (4.8)
Funding	No funding noted	31 (79.5)	26 (61.9)
	Federal	4 (10.3)	5 (11.9)
	Private	4 (10.3)	11 (26.2)
	Funded, source unknown	3 (7.7)	0
Joint distribution	Knee	28 (66.7)	35 (83.3)
	Wrist/hand	7 (16.7)	1 (2.4)
	Hip	2 (4.8)	1 (2.4)
	Multiple joints	1 (2.4)	3 (7.1)
	Shoulder	2 (4.8)	2 (4.8)
	Foot/ankle	2 (4.8)	0
KL grade	KL 1	313 (12.4)	262 (10.3)
	KL 2	586 (23.2)	958 (37.7)
	KL 3	814 (32.2)	967 (38.0)
	KL 4	818 (32.3)	356 (14.0)
US guidance	Yes	16 (38.1)	22 (52.4)
	No	15 (35.7)	12 (28.6)
	Unknown	11 (26.2)	8 (19.0)
Study follow‐up	≤1 y	24 (57.1)	35 (83.3)
	>1 y	18 (42.9)	7 (16.7)
Preintervention protocol	Yes	1 (2.4)	11 (26.2)
	No	41 (97.6)	31 (73.8)
Postintervention protocol	Yes	21 (50)	15 (35.7)
	No	21 (50)	27 (64.3)

Abbreviations: BMAC, bone marrow aspirate concentrate; KL, Kellgren–Lawrence; RCT, randomized controlled trial; US, ultrasound.

### Population characteristics

Gender of participants was reported in 92% (*n* = 77) of studies, with females comprising 56% of the study participants across studies. In the 47 studies that reported age (56% of studies), the average age was 58.6 ± 8.5 years (mean ± SD). Only 30% (*n* = 26) of the studies reported body mass index (BMI); the average reported BMI in these studies was 26.7 ± 2 (mean ± SD). Only six studies provided information on race and ethnicity. Further details are included in Table S2.

### Follow‐up period

The follow‐up varied from 6 weeks to 5 years, with an average study duration of 16.3 ± 9 months. Overall, 64% (*n* = 27) of the BMAC studies^29^, ^42^, ^44^, ^45^, ^46^, ^48^, ^52^, ^53^, ^60^, ^61^, ^67^, ^72^, ^74^, ^80^, ^84^, ^85^, ^92^, ^96^, ^97^, ^99^, ^102^, ^106^, ^111^, ^112^ and 50% (*n* = 21) of the adipose studies,^32^, ^34^, ^35^, ^36^, ^38^, ^43^, ^49^, ^55^, ^56^, ^62^, ^64^, ^66^, ^68^, ^71^, ^76^, ^83^, ^90^, ^93^, ^104^, ^110^ representing more than half of all studies analyzed, reported a follow‐up period of exactly 12 months. Table 2 dichotomizes the studies in <1 year and >1 year.

### Cellular treatment modality

Half of the studies (*n* = 42) used BMAC^29^, ^30^, ^32^, ^33^, ^39^, ^42^, ^44^, ^45^, ^46^, ^47^, ^48^, ^51^, ^52^, ^53^, ^57^, ^60^, ^61^, ^67^, ^74^, ^75^, ^78^, ^80^, ^81^, ^84^, ^85^, ^89^, ^92^, ^94^, ^96^, ^97^, ^98^, ^99^, ^100^, ^101^, ^102^, ^103^, ^105^, ^106^, ^111^, ^112^ and the other half (*n* = 42) used adipose‐derived treatment^31^, ^34^, ^35^, ^36^, ^37^, ^38^, ^40^, ^41^, ^43^, ^49^, ^54^, ^55^, ^56^, ^58^, ^59^, ^62^, ^63^, ^64^, ^65^, ^66^, ^68^, ^69^, ^70^, ^71^, ^73^, ^76^, ^77^, ^79^, ^82^, ^83^, ^86^, ^87^, ^88^, ^90^, ^91^, ^93^, ^95^, ^104^, ^107^, ^108^, ^109^, ^110^. The studies reviewed were published between 2014 and 2024, with most of the studies published in 2022.

### Patient‐reported outcomes scores

Either the visual analogue scale or numeric pain scale was used in 77% of studies (*n* = 65) to assess patient pain. Knee Injury and Osteoarthritis Outcomes Score (KOOS) was the most used patient‐reported outcome measure (PROM), followed by Western Ontario McMaster Universities Osteoarthritis Index (WOMAC) and International Knee Documentation Committee in 32% (*n* = 27), 22% (*n* = 19), and 13% (*n* = 11) studies, respectively. More than one PROM was used in 33% of the studies (*n* = 28). Out of the 14 studies involving the upper extremity, 6 used the Disabilities of the Arm, Shoulder and Hand Questionnaire (DASH), 3 the Quick DASH, 2 the Oxford Shoulder Score, 1 the Shoulder Simple Score, 1 the Michigan Hand Outcomes Score, and 1 the Western Ontario Osteoarthritis of the Shoulder Index. Further details are included in Table S1.

### Imaging outcomes

Imaging outcomes were reported in limited studies, with 10% of the studies (*n* = 9) using magnetic resonance imaging (MRI) as the follow‐up tool to evaluate response to treatment^41^, ^43^, ^70^, ^75^, ^79^, ^96^, ^97^, ^108^, ^112^ and 2% (*n* = 2) using conventional radiography.^58^, ^85^

### Severity of osteoarthritis

The severity of OA was reported in 64% (*n* = 53) of the studies that used the Kellgren–Lawrence (KL) scale^112^ and described 5074 total joints. The majority of these were knee studies (*n* = 36), with seven hand/wrist, five shoulder, two hip, two foot/ankle, and one study with knee and shoulder detailing the KL scores (Table S2). Of the joints detailed, 11% (*n* = 575) were classified as KL1, 30% (*n* = 1544) as KL2, 35% (*n* = 1781) KL3, and 23% (*n* = 1174) as KL4 (Table 2). In general, adipose studies included more severe OA than BMAC studies (Table S2).

### Injection characteristics

More than half (51%) of studies (*n* = 43) reported that participants received the same volume of injectate for each treatment,^29^, ^30^, ^32^, ^33^, ^34^, ^35^, ^37^, ^39^, ^43^, ^50^, ^52^, ^55^, ^57^, ^60^, ^61^, ^66^, ^67^, ^68^, ^69^, ^71^, ^72^, ^73^, ^77^, ^78^, ^79^, ^81^, ^86^, ^87^, ^88^, ^89^, ^91^, ^93^, ^94^, ^95^, ^96^, ^97^, ^98^, ^101^, ^103^, ^104^, ^106^, ^107^, ^109^ 30% (*n* = 25) reported a range of volumes,^31^, ^40^, ^45^, ^46^, ^47^, ^51^, ^53^, ^54^, ^56^, ^62^, ^64^, ^65^, ^70^, ^74^, ^75^, ^82^, ^84^, ^85^, ^92^, ^99^, ^100^, ^102^, ^105^, ^108^, ^112^ and 19% (*n* = 16) did not report any volume information.^36^, ^38^, ^41^, ^42^, ^44^, ^48^, ^49^, ^58^, ^59^, ^63^, ^76^, ^80^, ^83^, ^90^, ^110^, ^111^ Only two studies reported a protocol with repeated injections^40^, ^59^ (Table S3).

Description of the injectate was included in 31% of studies (*n* = 25) reporting on cell characterization.^29^, ^30^, ^35^, ^36^, ^37^, ^45^, ^46^, ^48^, ^50^, ^51^, ^57^, ^60^, ^61^, ^67^, ^71^, ^74^, ^75^, ^80^, ^84^, ^85^, ^96^, ^97^, ^106^, ^111^, ^112^ The strategies for analyses varied, with some authors testing all samples, others using nonrandom (*n* = 21)^35^, ^36^, ^37^, ^44^, ^45^, ^48^, ^50^, ^57^, ^60^, ^61^, ^67^, ^71^, ^74^, ^75^, ^80^, ^84^, ^85^, ^96^, ^97^, ^106^, ^112^ or random sampling of the injectates (*n* = 4).^29^, ^30^, ^51^, ^111^ There was inconsistent reporting on data presented, with quantified cellular dose provided in 24% of these studies (*n* = 5), all describing BMAC therapy.^45^, ^74^, ^84^, ^85^, ^112^

### Image guidance

Ultrasound guidance was used to deliver the injection in 45% (*n* = 38) of the studies,,^29^, ^30^, ^35^, ^37^, ^40^, ^42^, ^44^, ^45^, ^46^, ^47^, ^48^, ^52^, ^53^, ^56^, ^58^, ^64^, ^65^, ^73^, ^78^, ^79^, ^80^, ^81^, ^84^, ^86^, ^87^, ^90^, ^94^, ^96^, ^97^, ^98^, ^99^, ^100^, ^103^, ^104^, ^110^ and 33% (*n* = 28) did not use image guidance.^32^, ^34^, ^39^, ^43^, ^50^, ^54^, ^57^, ^60^, ^61^, ^62^, ^67^, ^68^, ^69^, ^74^, ^75^, ^76^, ^77^, ^82^, ^83^, ^91^, ^93^, ^105^, ^106^, ^107^, ^109^, ^111^ The remaining studies (22%, *n* = 18) did not specify whether image guidance was used.^31^, ^33^, ^38^, ^41^, ^49^, ^51^, ^55^, ^59^, ^70^, ^71^, ^72^, ^85^, ^89^, ^92^, ^95^, ^101^, ^108^, ^112^ Further details are included in Table S1.

#### Outcomes

Reported clinical outcomes of cellular therapies relative to controls in RCTs demonstrated mixed efficacy. For adipose‐derived cell therapies, four of the nine RCTs reported superior outcomes compared with controls, including three trials versus PRP^49^, ^79^, ^107^ and one versus corticosteroid or saline.^93^ The remaining five adipose RCTs demonstrated no significant difference when compared with PRP.^35^, ^36^, ^37^, ^39^, ^76^, ^113^

Similar findings were observed in BMAC studies; 40% of RCTs reported superior outcomes relative to control treatments. However, one of these trials compared different concentrations of BMAC rather than a separate control intervention.^111^ Of the four trials demonstrating better outcomes, two compared BMAC with hyaluronic acid,^39^, ^60^ one with corticosteroid injection,^52^ and one with exercise alone, although the experimental arm used BMAC combined with PRP and platelet lysate.^47^ The remaining studies demonstrated no significant difference, including two versus PRP,^29^, ^30^ one versus multiple orthobiologic comparators (SVF, umbilical cord stem cells, and corticosteroid),^80^ and two versus saline.^96^, ^97^

In contrast, when all studies were assessed in comparison to baseline, 98% (*n* = 81) reported statistically significant changes.^29^, ^30^, ^31^, ^32^, ^34^, ^35^, ^37^, ^38^, ^39^, ^40^, ^41^, ^42^, ^43^, ^44^, ^45^, ^47^, ^48^, ^49^, ^50^, ^51^, ^52^, ^53^, ^54^, ^55^, ^56^, ^57^, ^58^, ^59^, ^60^, ^61^, ^62^, ^63^, ^64^, ^65^, ^66^, ^67^, ^68^, ^69^, ^70^, ^71^, ^72^, ^73^, ^74^, ^75^, ^76^, ^77^, ^78^, ^79^, ^80^, ^81^, ^82^, ^83^, ^84^, ^85^, ^86^, ^87^, ^88^, ^89^, ^90^, ^91^, ^92^, ^93^, ^94^, ^95^, ^96^, ^97^, ^98^, ^99^, ^100^, ^101^, ^102^, ^103^, ^104^, ^105^, ^106^, ^107^, ^108^, ^110^, ^111^, ^112^, ^113^, ^114^

### Safety

A form of adverse events was reported in 77% of the studies (*n* = 65). The remaining 24% (*n* = 20) did not mention adverse events (Table S3).

### Pre‐ and postprocedure protocols

The pre‐ and postprocedure protocols were focused primarily on medications to avoid before and after the procedure and activity level post intervention, respectively. Preprocedure use of anti‐inflammatory medications was restricted in nine studies.^29^, ^30^, ^37^, ^45^, ^46^, ^48^, ^101^, ^103^

Following injection, 33% of the studies (*n* = 28) described NSAID use with nine allowing use immediately^35^, ^36^, ^37^, ^95^, ^96^, ^97^, ^109^ and  19 limiting NSAID use for at least 1 week after intervention.^29^, ^30^, ^58^, ^59^, ^64^, ^67^, ^74^, ^75^, ^77^, ^79^, ^83^, ^84^, ^85^, ^90^, ^94^, ^101^, ^102^, ^107^, ^112^

Weight‐bearing status was described in 62% of studies (*n* = 52), with 79% (*n* = 41) allowing full weight bearing, 15% (*n* = 8) partial weight bearing, and 6% (*n* = 3) nonweight bearing. Physical therapy protocol was described in 4% of studies (*n* = 3) (Table 2; Table S3).

## DISCUSSION

To our knowledge, this is the first scoping review to examine CBI for the treatment of OA across all joints in adult patients, limited to products compliant with current FDA guidance in the United States as of 2026, a specification that excludes treatments such SVF and culture‐expanded products. Previously published scoping reviews of CBI for OA have been limited to the hip joint^40^ or SVF,^44^ which is prohibited for clinical use in the United States outside of FDA‐approved trials.

The objective of this study was to synthesize research on CBI and identify research gaps to help inform future investigation in this area. The key findings are the limited number of RCTs, divergent cell preparation and injection methodologies, and variance in techniques to assess response to the interventions.

### Study design and follow‐up

Of the 84 included studies, only 19 were RCTs, with most other included studies being either prospective or retrospective cohorts. [Correction added on 16 May 2026 after first online publication: The previous statement has been revised.] Only 30% of all studies followed patients for >1 year. With the costs and risks associated with CBI including the cell harvest, long‐term results for pain and functional improvement superior to PRP are expected by the medical community and lay population. Although PRP with its multitude of growth factors affects cell adhesion, migration, and proliferation, evidence suggests that MSCs with enhanced immunomodulatory effect and higher interleukin 1 receptor antagonist concentration^114^ could potentially have a superior clinical response.

An important aspect to be noted is the limited use of imaging guidance for these injectable treatments. It was implemented in only 55% of the studies. A Delphi consensus outlined the need for guidance when injecting cellular products for precision and optimal care.^115^

### CBI and joint studied

There is a substantial gap in the literature regarding CBI for joints such as hips, shoulders, and wrists. The knee has the largest number of the studies and it is known to be a complacent joint, receiving up to 40 mL of fluid in an arthrogram.^116^ For this reason, it could receive more volume for optimal response. However, that is not the case for joints like the hip, hand, and wrist. The excessive volume can be uncomfortable.^117^ Furthermore, it is not fully understood in the setting of orthobiologics how structure, load, and biomechanics affect the efficacy of these treatments in other joints. There appears to be an optimal dose response based on the number of cells injected.^45^, ^112^, ^118^ However, different volumes are injected in the hip, hand, and wrist. Therefore, a consistent individualized approach per joint to establish the minimum number of cells injected is required.

### Standardized reporting – Demographics and phenotype

Patient characteristics, such as gender, race, age, and BMI, as well as phenotypic detail about the level of OA (KL grade), are known to influence outcomes but were infrequently reported. Only 30% of included studies provided BMI, 7% included race and ethnicity, and 64% reported KL grades. Similar to corticosteroid, hyaluronic acid, and PRP injections, CBI tends to provide better pain and functional outcomes in patients with lower grade OA.^104^, ^119^, ^120^, ^121^, ^122^ Including diverse demographic data points and phenotypic detail such as KL grade or other measures like MRI osteoarthritis knee score or ultrasound or MRI evidence of synovitis are essential to establish OA responder versua nonresponder phenotypes.^123^

### Standardized reporting – Type of cellular based injections

The included studies used a wide range of CBI. We have characterized them broadly as bone marrow aspirate and adipose‐derived products; however, they include BMAC, bone marrow aspirate (unconcentrated bone marrow aspirate), and adipose tissue. Pertaining to adipose tissue, each manufacturer has its unique way of preparing the fat for injection and to date it is unclear which devices provide a higher number of MSCs. For the field to evolve, the goal should be to standardize harvest and processing techniques with clear reporting for reproduction and clinical transparency.

### Cell quantification and dosing response

With most literature suggesting that higher injectate MSC concentrations lead to superior clinical^84^, ^119^, ^120^ and possibly radiological results,^83^ efforts toward consistently reporting injectate characteristics are ideal. In this review, only 25 studies reported some characterization, with 6 of them reporting dosing protocols assessing the impact of cell count on patient‐reported outcomes. Total nucleated cell counts can be measured at bedside, but they do not directly correlate with colony forming units (CFUs),^121^ which better estimate the desired MSCs. Furthermore, the techniques to complete a bone marrow aspiration and a fat harvest are essential for retrieval of a higher number of cells. For bone marrow, multiple smaller draws of 10 mL syringes and harvesting from both posterior superior iliac spines will render a better aspirate.^124^, ^125^ Use of ultrasound guidance for aspiration in bone marrow^126^ and microfragmented fat^127^ could further enhance the amount of tissue collected. Further research is required in the adipose arena to determine optimal fat harvest technique.

Although valuable, routine measurements of detailed cellular information are challenging to perform outside of a specialized center due to cost and required personnel. This creates a gap but also an opportunity for the development of point‐of‐care devices that can efficiently and reliably calculate the number of MSCs injected and enable tailored treatments based on cell counts. This will further establish a dose–response curve. At a minimum, studies should report injection volume and the concentrating device used. The optimal standard would be analysis of CFUs. Looking ahead, this will allow development of guidelines that recommend volumes, cell counts, and CFU based on the type of injected graft (BMAC, MFAT, etc).

### Pre‐ and postinjection protocols

Pre‐ (17%, *n* = 14) and post‐ (33%, *n* = 28) injection protocols were infrequently reported; however, all upper extremity studies provided protocols. This reporting inconsistency has been a long‐standing concern in orthobiologic publications. The ongoing question of whether NSAID use affects orthobiologic treatment outcomes remains unanswered and is not supported by clinical evidence but rather by theory, expert opinion, and in vitro findings.^128^ This may result in nonevidence‐based recommendations based on the design of the included studies. Establishing a consensus plan for periprocedural protocols would streamline these recommendations and allow for investigation into their effectiveness.^129^

### Consistency in PROM use and minimal clinically important difference

There is heterogeneity in the selection of the PROMs, which makes comparison across studies difficult. These findings illustrate the need for cross‐comparison of outcomes measures. It is essential to establish particular PROMs to be used for specific joints and to define minimal clinically important difference to assess the true impact of interventions. There is a need to assess statistically significant, meaningful clinical improvement that will positively affect joint function and quality of life measures^130^ and this can be achieved only if minimal clinically important difference is a routine assessment.

### Imaging protocols and outcomes

There were also limited imaging data reported to assess change in articular cartilage after CBI treatment. Only nine studies^41^, ^43^, ^70^, ^75^, ^79^, ^96^, ^97^, ^108^, ^112^ reported pre‐ and posttreatment MRI with a diversity of outcome measures and protocols. Similar to PROMs, this heterogeneity in imaging protocols and outcomes makes comparison difficult. Assessment of other tissues with advanced imaging may provide deeper insights on the effectiveness of CBI. Synovitis is a novel factor that could play a role in OA initiation, progression, and response to treatment. Emerging evidence suggests that synovitis on ultrasound or MRI may be a positive prognostic indicator in patients receiving orthobiologics injections.^131^ However, imaging‐based prognostic research remains limited for other forms of OA, including posttraumatic OA.^132^ Standardized recommendations for imaging protocols when evaluating articular cartilage, joint space, or synovial tissue, with different imaging modalities, would allow greater cross‐comparison of outcomes and perhaps effectiveness of CBI.^133^

### Limitations

The limitations are inherent to a scoping review. These include the lack of outcome reporting and comparison and the lack of risk of bias analysis of the included studies, which may have given equal weight to studies of varying quality.

### Conclusions

The results of this scoping review indicate the strong interest in and application of CBI across a broad spectrum of settings and patients. The review also highlights multiple areas for future focus to address identified research gaps. Future research designs should focus on RCTs, with an effort to expand to joints beyond the knee, which accounts for nearly all currently available high‐quality evidence. Further expectations for design improvements include standardizing the use of imaging guidance for injection procedures; ensuring clear and consistent documentation of patient characteristics including the OA phenotype with KL grading or other standard measures, as well as consistent reporting of patient characteristics to include age, gender, race, BMI; and documenting the volume and content of injectate to allow further analysis of such variables as cell volume, CFUs, and MSCs. A move toward standardized outcome measures would benefit comparisons across different studies. In conjunction with standardized outcome measures, data capture should extend to a period of at least 12 months or longer to demonstrate whether long‐term pain and function benefits exist and also to answer questions of potential regenerative effects. Lastly, the clear documentation of pre‐ and postprocedure protocols will allow the development of evidence‐based protocols that will aid patient recovery and lead to more successful outcomes in the future.

## FUNDING INFORMATION

None.

## DISCLOSURE

Leonardo Oliveira, Consultant: Trice Medical, Lipogems. Kenneth Mautner, Consultant: Lipogems.

## Supporting information


**Table S1.** Summary of studies included in analysis

**Table S2.** Basic demographics of studies included in the analysis

**Table S3.** Summary of study characteristics included in the analysis

## Data Availability

The data that support the findings of this study are available in the Supporting Information of this article and are available from the corresponding author upon reasonable request.
