# Supplementary material for: Adipose‐derived and bone marrow aspirate concentrate injections for osteoarthritis: A scoping review
Source: PM R. 2026 May 2;18(Suppl 2):S6–S19. doi: 10.1002/pmrj.70147 (PMC13193529; doi:10.1002/pmrj.70147)
Supplement: Supplementary file 1 — Table S1. Summary of studies included in analysis [file PMRJ-18-S6-s003.docx]

| **First Author** | **Year** | **Title** | **Study Type** | **Country** | **Joint** | **Cellular Therapy** | **Comparison group** | **Funding type** | **Patient-Reported Outcome Measures** |
| --- | --- | --- | --- | --- | --- | --- | --- | --- | --- |
| Anz | 2020 | Bone Marrow Aspirate Concentrate Is Equivalent to Platelet-Rich Plasma for the Treatment of Knee Osteoarthritis at 1 Year | RCT | United States | Knee | BMAC | PRP | Private | IKDC, WOMAC |
| Anz | 2022 | Bone Marrow Aspirate Concentrate Is Equivalent to Platelet-Rich Plasma for the Treatment of Knee Osteoarthritis at 2 Years | RCT | United States | Knee | BMAC | LP-PRP | Private | IKDC, WOMAC |
| Baek | 2024 | Effectiveness and Complications of Bone Marrow Aspirate Concentrate in Patients with Knee Osteoarthritis of Kellgren-Lawrence Grades II-III | Retrospective | South Korea | Knee | BMAC |  | No funding noted | VAS |
| Baek | 2024 | Comparison of Pain and Complications between Outpatients and Inpatients Treated with Bone Marrow Aspirate Concentrate for Knee Osteoarthritis | Retrospective | South Korea | Knee | BMAC (local anesthesia) | BMAC (local and general anesthesia) | No funding noted | KSS, VAS |
| Bąkowski | 2021 | Patients with stage II of the knee osteoarthritis most likely benefit from the intra-articular injections of autologous adipose tissue from 2 years of follow-up studies | Retrospective | Poland | Knee | ADIPOSE |  | No funding noted | EQ-5D, KOS, NRS, WOMAC |
| Barfod | 2019 | Treatment of osteoarthritis with autologous and microfragmented adipose tissue | Prospective | Denmark | Knee | ADIPOSE |  | No funding noted | KOOS |
| Baria | 2022 | Platelet-Rich Plasma Versus Microfragmented Adipose Tissue for Knee Osteoarthritis | RCT | United States | Knee | ADIPOSE | PRP | Private | KOOS, Tegner, VAS |
| Baria | 2024 | Microfragmented Adipose Tissue Is Equivalent to Platelet-Rich Plasma for Knee Osteoarthritis at 12 Months Posttreatment | RCT | United States | Knee | ADIPOSE | PRP | Private | KOOS, Tegner, VAS |
| Baria | 2024 | Relationship of Body Mass Index on Patient-Reported Outcomes After Platelet-Rich Plasma Versus Microfragmented Adipose Tissue for Knee Osteoarthritis | RCT | United States | Knee | ADIPOSE | PRP | No funding noted | KOOS |
| Bayram | 2024 | Intra-articular injection of autologous fat tissue in the treatment of patients with chronic knee pain due to osteoarthritis | Retrospective | Turkey | Knee | ADIPOSE |  | No funding noted | OKS, WOMAC, VAS |
| Boffa | 2022 | Bone marrow aspirate concentrate injections provide similar results versus viscosupplementation up to 24 months of follow-up in patients with symptomatic knee osteoarthritis. A randomized controlled trial | RCT | Italy | Knee | BMAC | HA | No funding noted | KOOS, VAS |
| Borg | 2021 | Gender-Specific Response in Pain and Function to Biologic Treatment of Knee Osteoarthritis: A Gender Bias-Mitigated, Observational, Intention-to-Treat Study at Two Years | Prospective | UK | Knee | ADIPOSE |  | No funding noted | OKS, VAS |
| Borić | 2019 | A 24-Month Follow-Up Study of the Effect of Intra-Articular Injection of Autologous Microfragmented Fat Tissue on Proteoglycan Synthesis in Patients with Knee Osteoarthritis | Prospective | Croatia | Knee | ADIPOSE |  | No funding noted | VAS |
| Burnham | 2021 | The safety and effectiveness of bone marrow concentrate injection for knee and hip osteoarthritis: a Canadian cohort | Prospective | Canada | Knee; Hip | BMAC |  | No funding noted | PDQQ, NRS |
| Castellarin | 2020 | Intra-articular administration of purified autologous adipose tissue for knee osteoarthritis treatment | Prospective | Italy | Knee | ADIPOSE |  | No funding noted | WOMAC, VAS |
| Centeno | 2014 | Efficacy of Autologous Bone Marrow Concentrate for Knee Osteoarthritis with and without Adipose Graft | Registry | United States | Knee | BMAC | BMC+PRP+Lipoaspirate | No funding noted | LEFS, NPS, SANE |
| Centeno | 2015 | A dose response analysis of a specific bone marrow concentrate treatment protocol for knee osteoarthritis | Retrospective | United States | Knee | BMAC | BMAC, PRP, PL | No funding noted | IKDC, Lower extremity function scale, NPS, SANE |
| Centeno | 2015 | A prospective multi-site registry study of a specific protocol of autologous bone marrow concentrate for the treatment of shoulder rotator cuff tears and osteoarthritis | Retrospective | United States | Shoulder | BMAC |  | No funding noted | DASH, NPS |
| Centeno | 2016 | A multi-center analysis of adverse events among 2372 adult patients undergoing adult autologous stem cell therapy for orthopaedic conditions | Registry | United States | Multiple joints | BMAC | BMAC + adipose graft, Culture expanded MSCs | No funding noted | None |
| Centeno | 2018 | A specific protocol of autologous bone marrow concentrate and platelet products versus exercise therapy for symptomatic knee osteoarthritis: a randomized controlled trial with 2 year follow-up | RCT | United States | Knee | BMAC | Physical therapy | Private | KSS |
| Dallo | 2021 | A Prospective Study Comparing Leukocyte-Poor  Platelet-Rich Plasma Combined with Hyaluronic Acid  and Autologous Microfragmented Adipose Tissue  in Patients with Early Knee Osteoarthritis | RCT | Multiple countries represented | Knee | ADIPOSE | LP-PRP + HA | No funding noted | KOOS, MARS, Tegner, VAS |
| Daoudi | 2021 | Intra-articular injection of autologous bone marrow aspirate concentrate in the treatment of osteoarthritis of the thumb first carpometacarpal joint: a pilot study | Retrospective | France | Hand/wrist | BMAC |  | No funding noted | NRS, PRWE, Quick DASH |
| Dulic | 2020 | Do knee injection portals affect clinical results of bone marrow aspirate concentrate injection in the treatment of osteoarthritis? A prospective randomized controlled study | RCT | Serbia | Knee | BMAC | BMAC (Different knee injection portals) | Federal | IKDC, KOOS, VAS |
| Dulic | 2021 | Bone Marrow Aspirate Concentrate versus Platelet Rich Plasma or Hyaluronic Acid for the Treatment of Knee Osteoarthritis | Prospective | Serbia | Knee | BMAC | PRP, HA | Federal | IKDC, KOOS, WOMAC, VAS |
| Dwyer | 2021 | Injection of Bone Marrow Aspirate for Glenohumeral Joint Osteoarthritis: A Pilot Randomized Control Trial | RCT | Canada | Shoulder | BMAC | Cortisone | Private | EQ-5, WOOS, VAS |
| El-Kadiry | 2022 | Bone marrow aspirate concentrate versus platelet-rich plasma for treating knee osteoarthritis: a one-year non-randomized retrospective comparative study | Retrospective | Canada | Knee | BMAC | PRP | Private | KOOS, WOMAC, VAS |
| Erne | 2018 | Autologous Fat Injection versus Lundborg Resection Arthroplasty for the Treatment of Trapeziometacarpal Joint Osteoarthritis | Retrospective | Germany | Hand/wrist | ADIPOSE | Lundborg resection arthroplasty | No funding noted | DASH, VAS |
| Estrada | 2020 | Patient-Reported Outcomes After Platelet-Rich Plasma, Bone Marrow Aspirate, and Adipose-Derived Mesenchymal Stem Cell Injections for Symptomatic Knee Osteoarthritis | Prospective | Argentina | Knee | ADIPOSE | BMAC, PRP | No funding noted | IKDC, KSS |
| Fan | 2022 | An Observational Study Evaluating the Efficacy of Microfragmented Adipose Tissue in the Treatment of Osteoarthritis | Prospective | UK | Knee; Shoulder | ADIPOSE | ADIPOSE | No funding noted | OKS, OSS, QuickDash, Tegner, VAS |
| Garay-Mendoza | 2018 | The effect of intra-articular injection of autologous bone marrow stem cells on pain and knee function in patients with osteoarthritis | Prospective | Mexico | Knee | BMAC | Acetaminophen | No funding noted | WOMAC, VAS |
| Gobbi | 2021 | Two-year clinical outcomes of autologous microfragmented adipose tissue in elderly patients with knee osteoarthritis: a multi-centric, international study | Retrospective | Italy, USA, UAE | Knee | ADIPOSE |  | No funding noted | KOOS, VAS |
| Gobbi | 2023 | Autologous microfragmented adipose tissue and leukocyteâ€‘poor plateletâ€‘rich plasma combined with hyaluronic acid show comparable clinical outcomes for symptomatic early knee osteoarthritis over a twoâ€‘year followâ€‘up period: a prospective randomized clinical trial | Retrospective | Italy | Knee | ADIPOSE | LP-PRP + HA | Federal | KOOS, VAS |
| Goncars | 2017 | The comparison of knee osteoarthritis treatment with  single-dose bone marrow-derived mononuclear cells vs. hyaluronic acid injections | RCT | Latvia | Knee | BMAC | HA | No funding noted | KOOS, KSS |
| Goncars | 2019 | Treatment of Knee Osteoarthritis with Bone Marrow-Derived Mononuclear Cell Injection: 12-Month Follow-up | Prospective | Latvia | Knee | BMAC |  | Federal | KOOS, KSS |
| Haas | 2020 | One-Year Outcomes of Intraarticular Fat Transplantation for Thumb Carpometacarpal Joint Osteoarthritis: Case Review of 99 Joints | Prospective | Germany | Hand/wrist | ADIPOSE |  | No funding noted | Michigan Hand Outcomes, VAS |
| Haas-Lützenberger | 2024 | Clinical results of autologous fat transfer for basal thumb arthritis with a minimum of three years' follow-up | Retrospective | Germany | Hand/wrist | ADIPOSE |  | No funding noted | Michigan hand outcome score, NRS, QuickDASH |
| Heidari | 2020 | Patient-Centered Outcomes of Microfragmented Adipose Tissue Treatments of Knee Osteoarthritis: An Observational,Intention-to-Treat Study at Twelve Months | Prospective | UK | Knee | ADIPOSE |  | Funded, source unknown | EQ-5D, OKS, VAS |
| Heidari | 2021 | Microfragmented Adipose Tissue Injection (MFAT) May Be a Solution to the Rationing of Total Knee Replacement: A Prospective, Gender-Bias Mitigated, Reproducible Analysis at Two Years | Retrospective | UK | Knee | ADIPOSE |  | No funding noted | EQ-5D, OKS |
| Heidari | 2022 | Comparison of the Effect of MFAT and MFAT + PRP on Treatment of Hip Osteoarthritis: An Observational, Intention-to-Treat Study at One Year | Prospective | UK | Hip | ADIPOSE | Adipose + PRP | No funding noted | OHS, VAS |
| Hernigou | 2021 | Subchondral bone or intra-articular injection of bone marrow concentrate mesenchymal stem cells in bilateral knee osteoarthritis: what better postpone knee arthroplasty at fifteen years? A randomized study | Prospective | France | Knee | BMAC | BMAC (subchondral) | No funding noted | KSS, VAS |
| Herold | 2017 | Autologous Fat Transfer for Thumb Carpometacarpal Joint Osteoarthritis: A Prospective Study | Prospective | Germany | Hand/wrist | ADIPOSE |  | No funding noted | DASH, VAS |
| Holzbauer | 2022 | Liparthroplasty for Thumb Carpometacarpal Joint Osteoarthritis: A Case Series with Median 5 Years of Follow-Up | Retrospective | Austria | Hand/wrist | ADIPOSE |  | No funding noted | DASH, VAS |
| Hudetz | 2017 | The Effect of Intra-articular Injection of Autologous Microfragmented Fat Tissue on Proteoglycan Synthesis in Patients with Knee Osteoarthritis | Prospective | Croatia | Knee | ADIPOSE |  | No funding noted | VAS |
| Hudetz | 2019 | Early results of intra-articular micro-fragmented lipoaspirate treatment in patients with late stages knee osteoarthritis: a prospective study | Prospective | Croatia | Knee | ADIPOSE |  | No funding noted | KOOS, WOMAC, VAS |
| Hussein | 2021 | Bone Marrow Aspirate Concentrate Is More Effective Than Hyaluronic Acid and Autologous Conditioned Serum in the Treatment of Knee Osteoarthritis: A Retrospective Study of 505 Consecutive Patients | Retrospective | Slovenia | Knee | BMAC | Autologous conditioned serum, HA | Federal | WOMAC, VAS |
| Iacono | 2023 | Efficacy and Duration of Intra-Articular Autologous Micro-Fragmented Adipose Tissue in Athletes with Ankle Osteoarthritis: A 36-Month Follow-Up Study | Retrospective | Italy | Foot/ankle | ADIPOSE |  | No funding noted | AOFAS, FADI, VAS |
| Jeyaraman | 2024 | Autologous Bone Marrow Aspiration Concentrate (BMAC) Therapy for Primary Knee Osteoarthritis An Observational and Dose Escalation Study | Prospective | India | Knee | BMAC | BMAC, Saline | No funding noted | IKDC, VAS |
| Jeyaraman | 2024 | Bone Marrow Aspirate Concentrate for Treatment of Primary Knee Osteoarthritis: A Prospective, Single-Center, Non-randomized Study with 2-Year Follow-Up | RCT | India | Knee | BMAC |  | Private | IKDC, KOOS, WOMAC |
| Kaszyński | 2022 | Intra-Articular Injections of Autologous Adipose Tissue or Platelet-Rich Plasma Comparably Improve Clinical and Functional Outcomes in Patients with Knee Osteoarthritis | RCT | Poland | Knee | ADIPOSE | PRP, negative control | No funding noted | EQ-5D-5L, KOOS, WOMAC, VAS |
| Kim | 2020 | Intra-Articular Bone Marrow Aspirate Concentrate Injection in Patients with Knee Osteoarthritis | Retrospective | Republic of Korea | Knee | ADIPOSE |  | Federal | IKDC, KOOS, SF-36, Tegner, VAS |
| Kuebler | 2022 | Short-Term Efficacy of Using a Novel Low-Volume Bone Marrow Aspiration Technique to Treat Knee Osteoarthritis: A Retrospective Cohort Study | Retrospective | United States | Knee | BMAC |  | Private | PGIC, WOMAC, VAS |
| Louis | 2021 | Intra-Articular Injection of Autologous Microfat and Platelet-Rich Plasma in the Treatment of Knee Osteoarthritis: A Double-Blind Randomized Comparative Study | RCT | France | Knee | ADIPOSE | Adipose + PRP | Private | WOMAC, VAS |
| Mautner | 2019 | Functional Outcomes Following Microfragmented Adipose Tissue Versus Bone Marrow Aspirate Concentrate Injections for Symptomatic Knee Osteoarthritis | Retrospective | United States | Knee | BMAC | Adipose | No funding noted | EQOL, KOOS, VAS |
| Mautner | 2023 | Cell-based versus corticosteroid injections for knee pain in osteoarthritis: a randomized phase 3 trial | RCT | United States | Knee | BMAC | SVF, umbilical cord tissue, corticosteroid | Private | KOOS, VAS |
| Meyer-Marcotty | 2022 | Lipofilling in Osteoarthritis of the Finger Joints: Initial Prospective Long-Term Results | Prospective | Germany | Hand/wrist | ADIPOSE |  | No funding noted | DASH |
| Miles | 2022 | Conversion to Knee Arthroplasty Following Intra-Articular Injection of Microfragmented Adipose  Tissue in Patients with Knee Osteoarthritis | Retrospective | United States | Knee | ADIPOSE |  | Funded, source unknown | KOOS Jr, SF-12 |
| Muthu | 2024 | Effect of Cellular Dosage of Bone Marrow Aspiration Concentrate on the Radiological Outcomes in Knee Osteoarthritis: A Phase I Dose Escalation Study | Prospective | India | Knee | BMAC | BMAC (different doses) | No funding noted | VAS |
| Muthu | 2024 | Increased Cellular Dosage of Bone Marrow Aspiration Concentrate Does Not Translate to Increased Clinical Effectiveness in Knee Osteoarthritis: A Phase I Dose Escalation Study | Prospective | India | Knee | BMAC | BMAC (different doses) | No funding noted | KOOS, VAS |
| Muthu | 2024 | Obese Patients Do Not Benefit from Bone Marrow Aspiration Concentrate Injection for Knee Osteoarthritis: A Prospective Cohort Study of 68 Patients | Prospective | India | Knee | BMAC | BMAC (different doses) | No funding noted | KOOS, VAS |
| Natali | 2021 | The use of intra-articular injection of autologous micro-fragmented adipose tissue as pain treatment for ankle osteoarthritis: a prospective not randomized clinical study | Prospective | Italy | Foot/ankle | ADIPOSE |  | No funding noted | AOFAS, FADI, VAS |
| Natali | 2023 | Is intra-articular injection of autologous micro-fragmented adipose tissue effective in hip osteoarthritis? A three year follow-up | Prospective | Italy | Hip | ADIPOSE |  | No funding noted | VAS |
| Natali | 2023 | Efficacy and Long-Term Outcomes of Intra-Articular Autologous Micro-Fragmented Adipose Tissue in Individuals with Glenohumeral Osteoarthritis: A 36-Month Follow-Up Study | Retrospective | Italy | Shoulder | ADIPOSE |  | No funding noted | CMS, SANE, SST, VAS |
| Pabinger | 2024 | Intra-articular injection of bone marrow aspirate concentrate (mesenchymal stem cells) in KL grade III and IV knee osteoarthritis: 4 year results of 37 knees | Prospective | Austria | Knee | BMAC |  | No funding noted | IKDC, SF-36, WOMAC |
| Panchal | 2018 | Safety and Efficacy of Percutaneous Injection of Lipogems Micro-Fractured Adipose Tissue for Osteoarthritic Knees | Prospective | United States | Knee | ADIPOSE |  | No funding noted | KSS, LEAS, NRPS |
| Pintore | 2023 | Intra-articular injection of bone marrow aspirate concentrate (BMAC) or adipose-derived stem cells (ADSCs) for knee osteoarthritis: a prospective comparative clinical trial | Prospective | Italy | Knee | ADIPOSE | BMAC | No funding noted | KOOS, OKS, VAS |
| Rasovic | 2023 | The role of osteoarthritis severity, BMI and age on clinical efficacy of bone marrow aspirate concentrate in the treatment of knee osteoarthritis | Prospective | Serbia | Knee | BMAC |  | Federal | IKDC, KOOS, WOMAC |
| Richter | 2024 | Microfragmented Adipose Tissue Injection Reduced Pain Compared With a Saline Control Among Patients With Symptomatic Osteoarthritis of the Knee During 1-Year Follow-Up: A Randomized Controlled Trial | RCT | United States | Knee | ADIPOSE | Corticosteroid, saline | Funded, source unknown | KOOS, VAS |
| Rodriguez-Fontan | 2018 | Early Clinical Outcomes of Intra-Articular Injections of Bone Marrow Aspirate Concentrate for the Treatment of Early Osteoarthritis of the Hip and Knee: A Cohort Study | Prospective | United States | Knee; Hip | BMAC |  | No funding noted | WOMAC |
| Screpis | 2022 | Autologous Microfragmented Adipose Tissue for the Treatment of Knee Osteoarthritis: Real-World Data at Two Years Follow-Up | Prospective | Italy | Knee | ADIPOSE |  | Federal | KOOS, VAS |
| Shapiro | 2017 | A Prospective, Single-Blind, Placebo-Controlled Trial of Bone Marrow Aspirate Concentrate for Knee Osteoarthritis | RCT | United States | Knee | BMAC | Saline | Private | ICOAP, OARSI, VAS |
| Shapiro | 2019 | Quantitative T2 MRI Mapping and 12-Month Follow-up in a Randomized, Blinded, Placebo Controlled Trial of Bone Marrow Aspiration and Concentration for Osteoarthritis of the Knees | RCT | United States | Knee | BMAC | Saline | Private | ICOAP, VAS |
| Shaw | 2018 | Short-Term Outcomes in Treatment of Knee Osteoarthritis With 4 Bone Marrow Concentrate Injections | Retrospective | United States | Knee | BMAC |  | No funding noted | NPS, SANE |
| Silvestre | 2023 | Intra-articular Injection of Bone Marrow Concentrate for Treatment of Patellofemoral Osteoarthritis: Preliminary Results Utilizing an Ultrasound-Guided Marrow Harvesting Technique | Retrospective | France | Knee | BMAC |  | No funding noted | IKDC, WOMAC, VAS |
| Smith | 2023 | Derivation of a Clinical Decision Rule for a Bone Marrow Aspirate Concentrate Injection in Knee Osteoarthritis | Prospective | Canada | Knee | BMAC |  | No funding noted | Pain disability score |
| Themistocleous | 2018 | Effectiveness of a single intra-articular bone marrow aspirate concentrate (BMAC) injection in patients with grade 3 and 4 knee osteoarthritis | Retrospective | Greece | Knee | BMAC |  | No funding noted | NPS, OKS |
| Tsitsilianos | 2022 | Bone marrow aspirate injection for osteoarthritis of the hip; A pilot study | Retrospective | United States | Hip | BMAC |  | Private | HOOS-Jr, NRS |
| Varady | 2020 | Positive early clinical outcomes of bone marrow aspirate concentrate for osteoarthritis using a novel fenestrated trocar | Prospective | United States | Knee | BMAC |  | No funding noted | Tegner, KOOS, VAS |
| Vinet-Jones | 2020 | Clinical use of autologous micro-fragmented fat progressively restores pain and function in shoulder osteoarthritis | Prospective | United States | Shoulder | ADIPOSE |  | No funding noted | DASH, VAS |
| Vitali | 2022 | Bone Marrow Aspirate Concentrate vs Autologous Conditioned Serum in the Treatment of Knee Osteoarthritis | Prospective | Italy | Knee | BMAC |  | No funding noted | WOMAC, VAS |
| Wells | 2021 | Cellular and Clinical Analyses of Autologous Bone Marrow Aspirate Injectate for Knee Osteoarthritis: A Pilot Study | Prospective | United States | Knee | BMAC |  | No funding noted | KOOS-Jr, NRS |
| Winter | 2023 | Minimally Invasive Treatment of Trapeziometacarpal Osteoarthritis: Results of a Blinded Randomized Controlled Trial | RCT | Austria | Hand/wrist | ADIPOSE | Adipose+ PRP, PRP, saline | No funding noted | NRS, SF-36, QuickDASH |
| Yu | 2023 | Intra-Articular Injection of Autologous Micro-Fragmented Adipose Tissue for the Treatment of Knee Osteoarthritis: A Prospective Interventional Study | Prospective | China | Knee | ADIPOSE |  | Federal | HSS, KSS, VAS |
| Zaffagnini | 2022 | Microfragmented Adipose Tissue Versus Platelet-Rich Plasma for the Treatment of Knee Osteoarthritis | RCT | Italy | Knee | ADIPOSE | PRP | Private | EQ-5, KOOS, VAS |
| Zannoni | 2024 | Clinical results in patients affected by moderate-severe knee osteoarthritis and treated with microâ€‘fragmented adipose tissue: the therapeutic effects on symptomatology | Retrospective | Italy | Knee | ADIPOSE |  | No funding noted | KOOS, VAS |

**Appendix. Abbreviations**

| **Abbreviation** | **Definition** |
| --- | --- |
| ADSC(s) | Adipose-derived stem cell(s) |
| AOFAS | American Orthopaedic Foot & Ankle Society score |
| BMAC | Bone marrow aspirate concentrate |
| BMI | Body mass index |
| CMS | Constant–Murley score |
| DASH | Disabilities of the Arm, Shoulder and Hand questionnaire |
| EQ-5 | EuroQol 5-dimension questionnaire |
| EQ-5D | EuroQol 5-dimension questionnaire |
| EQ-5D-5L | EuroQol 5-dimension, 5-level questionnaire |
| EQOL | EuroQol quality of life |
| FADI | Foot and Ankle Disability Index |
| HA | Hyaluronic acid |
| HSS | Hospital for Special Surgery score |
| HOOS Jr | Hip disability and Osteoarthritis Outcome Score, Junior |
| ICOAP | Intermittent and Constant Osteoarthritis Pain |
| IKDC | International Knee Documentation Committee score |
| ITT | Intention-to-treat |
| KL | Kellgren–Lawrence grade |
| KOOS | Knee injury and Osteoarthritis Outcome Score |
| KOOS Jr | Knee injury and Osteoarthritis Outcome Score, Junior |
| KSS | Knee Society Score |
| LEAS | Lower Extremity Activity Scale |
| LEFS | Lower Extremity Functional Scale |
| LP-PRP | Leukocyte-poor platelet-rich plasma |
| MARS | Modified Activity Rating Scale |
| MFAT | Microfragmented adipose tissue |
| MSC(s) | Mesenchymal stem cell(s) |
| NPS | Numeric pain scale |
| NRS | Numeric rating scale |
| NRPS | Numeric rating pain scale |
| OARSI | Osteoarthritis Research Society International |
| OKS | Oxford Knee Score |
| OSS | Oxford Shoulder Score |
| PGIC | Patient Global Impression of Change |
| PL | Platelet lysate |
| PRP | Platelet-rich plasma |
| PRWE | Patient-Rated Wrist Evaluation |
| QuickDASH | Shortened Disabilities of the Arm, Shoulder and Hand questionnaire |
| RCT | Randomized controlled trial |
| SANE | Single Assessment Numeric Evaluation |
| SF-12 | Short Form Health Survey, 12-item |
| SF-36 | Short Form Health Survey, 36-item |
| SST | Simple Shoulder Test |
| SVF | Stromal vascular fraction |
| Tegner | Tegner Activity Scale |
| VAS | Visual analog scale |
| WOMAC | Western Ontario and McMaster Universities Osteoarthritis Index |
