# Supplementary material for: Adipose‐derived and bone marrow aspirate concentrate injections for osteoarthritis: A scoping review
Source: PM R. 2026 May 2;18(Suppl 2):S6–S19. doi: 10.1002/pmrj.70147 (PMC13193529; doi:10.1002/pmrj.70147)
Supplement: Supplementary file 2 — Table S2. Basic demographics of studies included in the analysis [file PMRJ-18-S6-s001.docx]

| **First Author** | **Year** | **Cellular Therapy** | **Joint** | **Number of patients** | **Female Sex (%)** | **Race/Ethnicity Reported?** | **Age (SD)** | **BMI (SD)** | **KL 1** | **KL 2** | **KL 3** | **KL 4** |
| --- | --- | --- | --- | --- | --- | --- | --- | --- | --- | --- | --- | --- |
| Anz | 2020 | BMAC | Knee | 84 | 35 (42%) | No |  |  |  |  |  |  |
| Anz | 2022 | BMAC | Knee | 84 | 35 (42%) | No |  |  |  |  |  |  |
| Baek | 2024 | BMAC | Knee | 120 | 96 (80%) | No |  |  |  | 66 | 54 |  |
| Baek | 2024 | BMAC | Knee | 231 | 172 (74%) | No | 64.2 (7.5) | 24.9 (3.2) |  | 86 | 199 |  |
| Bąkowski | 2021 | ADIPOSE | Knee | 37 | 21 (57%) | No | 57.8 (7.4) | 31.3 (7.5) | 1 | 9 | 20 | 7 |
| Barfod | 2019 | ADIPOSE | Knee | 20 |  | No | 49 (9) |  |  |  |  |  |
| Baria | 2022 | ADIPOSE | Knee | 58 | 30 (52%) | Yes |  |  | 8 | 13 | 23 | 14 |
| Baria | 2024 | ADIPOSE | Knee | 49 | 25 (51%) | Yes |  |  | 8 | 12 | 17 | 12 |
| Baria | 2024 | ADIPOSE | Knee | 49 | 25 (51%) | No |  |  | 8 | 12 | 17 | 12 |
| Bayram | 2024 | ADIPOSE | Knee | 165 | 103 (62%) | No | 61.3 (11.4) | 26.2 (4.5) | 17 | 58 | 64 | 26 |
| Boffa | 2022 | BMAC | Knee | 56 | 21 (38%) | No | 57.8 (8.9) | 27.8 (4.3) | 3 | 75 | 28 | 6 |
| Borg | 2021 | ADIPOSE | Knee | 386 | 192 (50%) | No | 65.5 |  | 13 | 73 | 92 | 208 |
| Borić | 2019 | ADIPOSE | Knee | 10 |  | No | 69 (12) |  |  |  |  |  |
| Burnham | 2021 | BMAC | Knee; Hip | 112 | 48 (43%) | No | 64.1 (9.1) |  |  |  |  |  |
| Castellarin | 2020 | ADIPOSE | Knee | 92 | 33 (36%) | No | 52 |  |  |  |  |  |
| Centeno | 2014 | BMAC | Knee | 681 | 324 (48%) | No |  |  |  |  |  |  |
| Centeno | 2015 | BMAC | Knee | 34 | 7(21%) | No | 52.1 (14.3) | 25.3 (2.9) |  |  |  |  |
| Centeno | 2015 | BMAC | Shoulder | 373 |  | No |  |  | 165 | 84 | 19 | 20 |
| Centeno | 2016 | BMAC | Multiple joints | 2372 | 931 (39%) | No | 55.6 (13.8) | 26.6 (4.7) |  |  |  |  |
| Centeno | 2018 | BMAC | Knee | 48 |  | No |  |  |  | 21 | 27 |  |
| Dallo | 2021 | ADIPOSE | Knee | 50 | 27 (54%) | No |  |  | 33 | 47 |  |  |
| Daoudi | 2021 | BMAC | Hand/wrist | 24 | 20 (83%) | No | 55 |  | 2 | 11 | 13 | 1 |
| Dulic | 2020 | BMAC | Knee | 111 | 51 (46%) | No |  |  |  | 49 | 46 | 16 |
| Dulic | 2021 | BMAC | Knee | 175 | 90 (51%) | No |  |  |  | 74 | 66 | 26 |
| Dwyer | 2021 | BMAC | Shoulder | 25 | 8 (32%) | No |  |  |  |  | 5 |  |
| El-Kadiry | 2022 | BMAC | Knee | 39 | 18 (46%) | No |  |  | 10 | 16 | 11 | 2 |
| Erne | 2018 | ADIPOSE | Hand/wrist | 21 | 17 (81%) | No |  |  |  |  |  |  |
| Estrada | 2020 | ADIPOSE | Knee | 89 |  | No |  |  |  |  |  |  |
| Fan | 2022 | ADIPOSE | Knee; Shoulder | 59 | 27 (46%) | No |  |  |  |  | 7 | 56 |
| Garay-Mendoza | 2018 | BMAC | Knee | 61 | 45 (74%) | No |  |  |  |  |  |  |
| Gobbi | 2021 | ADIPOSE | Knee | 75 | 49 (65%) | No | 69.6 (5.6) | 28.4 (5.3) |  | 18 | 68 | 34 |
| Gobbi | 2023 | ADIPOSE | Knee | 80 | 41 (51%) | No |  |  | 33 | 47 |  |  |
| Goncars | 2017 | BMAC | Knee | 56 | 31 (55%) | No |  |  |  | 16 | 40 |  |
| Goncars | 2019 | BMAC | Knee | 32 | 16 (50%) | No | 53.96 (14.2) |  |  | 16 | 18 |  |
| Haas | 2020 | ADIPOSE | Hand/wrist | 89 | 69 j (70%) | No | 61 (9.7) |  | 8 | 27 | 58 |  |
| Haas-Lützenberger | 2024 | ADIPOSE | Hand/wrist | 79 | 61 j (70%) | No | 61.2 | 24.4 (4.7) | 54 | 26 | 7 |  |
| Heidari | 2020 | ADIPOSE | Knee | 110 | 60 (55%) | No | 42-94 |  | 1 | 12 | 20 | 68 |
| Heidari | 2021 | ADIPOSE | Knee | 220 | 95 (43%) | No |  |  |  |  | 113 | 231 |
| Heidari | 2022 | ADIPOSE | Hip | 147 | 73 (50%) | No |  |  | 25 | 28 | 33 | 61 |
| Hernigou | 2021 | BMAC | Knee | 60 | 35 (58%) | No | 61 | 28.1 | 22 | 40 | 38 | 20 |
| Herold | 2017 | ADIPOSE | Hand/wrist | 50 | 38 (76%) | No | 59.9 |  |  | 25 | 18 | 7 |
| Holzbauer | 2022 | ADIPOSE | Hand/wrist | 31 | 27 (87%) | No | 57.7 | 27 (7) |  | 7 | 24 |  |
| Hudetz | 2017 | ADIPOSE | Knee | 17 | 5 (29%) | No | 69 (12) |  |  |  |  |  |
| Hudetz | 2019 | ADIPOSE | Knee | 20 | 5 (25%) | No |  |  |  |  |  |  |
| Hussein | 2021 | BMAC | Knee | 505 | 230 (46%) | No |  |  |  |  |  |  |
| Iacono | 2023 | ADIPOSE | Foot/ankle | 21 | 5 (24%) | No | 23.9 (4.5) | 26 (4.7) | 11 |  | 10 |  |
| Jeyaraman | 2024 | BMAC | Knee | 80 | 42 (53%) | No |  |  |  | 51 | 29 |  |
| Jeyaraman | 2024 | BMAC | Knee | 63 | 35 (56%) | No | 38.4 (12.2) | 30.2 (9.2) |  | 25 | 38 |  |
| Kaszyński | 2022 | ADIPOSE | Knee | 60 |  | No |  |  |  | 23 | 17 |  |
| Kim | 2020 | ADIPOSE | Knee | 25 | 16 (64%) | No | 67.5 | 26.3 (4.2) | 1 | 27 | 12 | 7 |
| Kuebler | 2022 | BMAC | Knee | 160 | 83 (52%) | No | 63.2 (1.0) |  |  |  |  |  |
| Louis | 2021 | ADIPOSE | Knee | 30 | 13 (43%) | No |  |  |  |  |  |  |
| Mautner | 2019 | BMAC | Knee | 76 | 40 (53%) | No |  |  |  |  |  |  |
| Mautner | 2023 | BMAC | Knee | 475 | 261 (55%) | Yes | 58.3 (7.7) | 30.8 (6.0) |  | 143 | 191 | 141 |
| Meyer-Marcotty | 2022 | ADIPOSE | Hand/wrist | 18 | 13 (72%) | No | 60.8  (46-76) |  |  | 8 | 12 | 5 |
| Miles | 2022 | ADIPOSE | Knee | 39 | 20 (5%) | No | 71.1  (47-95) | 28.4  (19-37) | 8 | 9 | 29 | 4 |
| Muthu | 2024 | BMAC | Knee | 75 | 40 (59%) | No |  |  | 20 | 48 |  |  |
| Muthu | 2024 | BMAC | Knee | 75 | 40 (59%) | No |  |  | 20 | 48 |  |  |
| Muthu | 2024 | BMAC | Knee | 68 | 40 (59%) | No |  |  | 20 | 48 |  |  |
| Natali | 2021 | ADIPOSE | Foot/ankle | 31 | 12 (39%) | No | 51.0 (15.5) |  | 3 | 15 | 13 |  |
| Natali | 2023 | ADIPOSE | Hip | 55 | 33 (60%) | No | 52.5 (10.9) | 23.7 (3.2) |  |  |  |  |
| Natali | 2023 | ADIPOSE | Shoulder | 65 | 32 (49%) | No | 54.2 (9.6) | 25 (3.4) | 53 | 7 | 5 |  |
| Pabinger | 2024 | BMAC | Knee | 29 | 11 (38%) | No | 58 (18) | 25 (18-35) |  |  | 15 | 22 |
| Panchal | 2018 | ADIPOSE | Knee | 17 | 7 (41%) | No | 68.3 (7.4) | 29 (4.5) |  |  | 7 | 19 |
| Pintore | 2023 | ADIPOSE | Knee | 102 | 56 (55%) | No |  |  |  |  |  |  |
| Rasovic | 2023 | BMAC | Knee | 111 | 54 (49%) | No |  |  |  | 9 | 46 | 16 |
| Richter | 2024 | ADIPOSE | Knee | 68 | 37 (54%) | No |  |  | 10 | 25 | 27 | 6 |
| Rodriguez-Fontan | 2018 | BMAC | Knee; Hip | 19 | 3 (16%) | Yes | 58 (12.7) | 25.9 (6) |  |  |  |  |
| Screpis | 2022 | ADIPOSE | Knee | 202 | 105 (52%) | No | 54.0 (9.0) | 26.8 (4.2) |  |  |  |  |
| Shapiro | 2017 | BMAC | Knee | 25 | 18 (72%) | Yes | 60  (42-68) | 27.1 |  | 11 | 12 | 2 |
| Shapiro | 2019 | BMAC | Knee | 25 | 18 (72%) | Yes | 60  (42-68) | 27.1 |  | 11 | 12 | 2 |
| Shaw | 2018 | BMAC | Knee | 15 | 10 (67%) | No | 67.7 (7.9) | 24.9 (2.7) |  |  |  |  |
| Silvestre | 2023 | BMAC | Knee | 96 | 41 (35%) | No |  |  |  |  |  |  |
| Smith | 2023 | BMAC | Knee | 93 | 43 (46%) | No | 64.1 (9.5) |  |  |  |  |  |
| Themistocleous | 2018 | BMAC | Knee | 121 | 85 (70%) | No | 70 |  |  |  | 46 | 75 |
| Tsitsilianos | 2022 | BMAC | Hip | 31 | 16 (52%) | No | 62.4 (16.5) |  |  | 10 | 14 | 7 |
| Varady | 2020 | BMAC | Knee | 17 | 10 (59%) | No | 54 |  |  |  |  |  |
| Vinet-Jones | 2020 | ADIPOSE | Shoulder | 25 |  | No |  |  |  | 12 | 13 |  |
| Vitali | 2022 | BMAC | Knee | 24 | 13 (54%) | No |  |  |  |  |  |  |
| Wells | 2021 | BMAC | Knee | 13 | 8 (62%) | No | 55.3 (7.0) |  |  |  |  |  |
| Winter | 2023 | ADIPOSE | Hand/wrist | 95 | 80 (81%) | No |  |  | 1 | 20 | 57 | 17 |
| Yu | 2023 | ADIPOSE | Knee | 20 | 12 (60%) | No | 55 (3.9) | 25.5 (2.86) |  |  |  |  |
| Zaffagnini | 2022 | ADIPOSE | Knee | 108 | 44 (41%) | No |  |  | 17 | 26 | 31 | 24 |
| Zannoni | 2024 | ADIPOSE | Knee | 38 |  | No | 62 (8.6) |  |  |  |  |  |

## **Appendix. Abbreviations**

| **Abbreviation** | **Definition** |
| --- | --- |
| ADIPOSE | Autologous adipose tissue–derived therapy |
| BMI | Body mass index |
| BMAC | Bone marrow aspirate concentrate |
| KL | Kellgren–Lawrence grade |
| SD | Standard deviation |
