# Supplementary material for: Adipose‐derived and bone marrow aspirate concentrate injections for osteoarthritis: A scoping review
Source: PM R. 2026 May 2;18(Suppl 2):S6–S19. doi: 10.1002/pmrj.70147 (PMC13193529; doi:10.1002/pmrj.70147)
Supplement: Supplementary file 3 — Table S3. Summary of study characteristics included in the analysis [file PMRJ-18-S6-s002.docx]

| **First Author** | | **Year** | **Celluar Therapy** | **Comparison Therapy** | **Joint** | **Number of Injections** | **Injectate Volume** | **Pre-Intervention Protocol** | **US Guidance** | **Post-Intervention protocol** | **Last Follow-up (months)** | **Adverse Events Reported** |
| --- | --- | --- | --- | --- | --- | --- | --- | --- | --- | --- | --- | --- |
|  | Anz | 2020 | BMAC | PRP | Knee | 1 | 7ml | Y | Y | Y | 12 | N |
|  | Anz | 2022 | BMAC | LP-PRP | Knee | 1 | 7ml | Y | Y | Y | 24 | N |
|  | Baek | 2024 | BMAC |  | Knee | 1 | 6ml | N | U | Y | 6 | Y |
|  | Baek | 2024 | BMAC | BMAC (local and general anesthesia) | Knee | 1 | 6 ml | N | N | Y | 12 | Y |
|  | Bąkowski | 2021 | ADIPOSE |  | Knee | 1 | not reported | N | U | N | 27 | Y |
|  | Barfod | 2019 | ADIPOSE |  | Knee | 1 | 10 ml | N | U | N | 12 | Y |
|  | Baria | 2022 | ADIPOSE | PRP | Knee | 1 | 5ml | Y | Y | Y | 6 | Y |
|  | Baria | 2024 | ADIPOSE | PRP | Knee | 1 | 5 ml | N | Y | Y | 12 | Y |
|  | Baria | 2024 | ADIPOSE | PRP | Knee | 1 | 5.2 ± 1.0 ml | N | N | Y | 12 | N |
|  | Bayram | 2024 | ADIPOSE |  | Knee | 1 | 7-10 ml | N | U | Y | 12 | Y |
|  | Boffa | 2022 | BMAC | HA | Knee | 1 | 6 ml | N | N | Y | 24 | Y |
|  | Borg | 2021 | ADIPOSE |  | Knee | 1 | not reported | N | Y | Y | 24 | Y |
|  | Borić | 2019 | ADIPOSE |  | Knee | 1 | 4-15 mL | N | U | N | 24 | N |
|  | Burnham | 2021 | BMAC |  | Knee; Hip | 1 | 8-10 ml | N | Y | Y | 12 | Y |
|  | Castellarin | 2020 | ADIPOSE |  | Knee | 1 | 6 ml | N | N | Y | 12 | N |
|  | Centeno | 2014 | BMAC | BMAC+PRP+Lipoaspirate | Knee | not reported | not reported | Y | Y | Y | 12 | Y |
|  | Centeno | 2015 | BMAC | BMAC, PRP, PL | Knee | 1 | not reported | Y | Y | Y | 12 | Y |
|  | Centeno | 2015 | BMAC |  | Shoulder | 1 | 10-15 ml | Y | Y | N | 12 | N |
|  | Centeno | 2016 | BMAC | BMAC + adipose graft, Culture expanded MSCs | Multiple joints | 1 | 1-3 ml | Y | Y | N | 12 | Y |
|  | Centeno | 2018 | BMAC | Physical therapy | Knee | 1 | 5-7 ml | Y | Y | Y | 34 | Y |
|  | Dallo | 2021 | ADIPOSE | LP-PRP + HA | Knee | 3 | 5ml | N | U | Y | 12 | Y |
|  | Daoudi | 2021 | BMAC |  | Hand/wrist | 1 | 2 ml | N | N | N | 26 | Y |
|  | Dulic | 2020 | BMAC | BMAC (Different knee injection portals) | Knee | 1 | not reported | N | No | Y | 12 | Y |
|  | Dulic | 2021 | BMAC | PRP, HA | Knee | 1 | 5-6 ml | N | U | N | 12 | Y |
|  | Dwyer | 2021 | BMAC | Cortisone | Shoulder | 1 | 10 ml | N | Y | Y | 12 | No |
|  | El-Kadiry | 2022 | BMAC | PRP | Knee | 1-3 | 15-43.75 ml | N | Y | N | 12 | N |
|  | Erne | 2018 | ADIPOSE | Lundborg resection arthroplasty | Hand/wrist | 1 | 1-2 ml | N | N | Y | 18 | Y |
|  | Estrada | 2020 | ADIPOSE | BMAC, PRP | Knee | 1 | 10 ml | N | U | N | 12 | N |
|  | Fan | 2022 | ADIPOSE | ADIPOSE | Knee; Shoulder | 1 | 6-8 ml | N | Y | N | 12 | N |
|  | Garay-Mendoza | 2018 | BMAC | Acetaminophen | Knee | 1 | 10 ml | Y | N | N | 6 | Y |
|  | Gobbi | 2021 | ADIPOSE |  | Knee | 1 | not reported | N | Y | Y | 24 | Y |
|  | Gobbi | 2023 | ADIPOSE | LP-PRP + HA | Knee | 3 | 5 ml | N | U | Y | 24 | Y |
|  | Goncars | 2017 | BMAC | HA | Knee | 1 | not reported | N | N | Y | 12 | Y |
|  | Goncars | 2019 | BMAC |  | Knee | 1 | 5ml | N | N | Y | 12 | Y |
|  | Haas | 2020 | ADIPOSE |  | Hand/wrist | 1 | 1-2 ml | N | N | Y | 12 | Y |
|  | Haas-Lützenberger | 2024 | ADIPOSE |  | Hand/wrist | 1 | not reported | N | N | Y | 36 | N |
|  | Heidari | 2020 | ADIPOSE |  | Knee | 1 | 6-8 ml | N | Y | Y | 12 | Y |
|  | Heidari | 2021 | ADIPOSE |  | Knee | 1 | 6-8 ml | N | Y | N | 24 | Y |
|  | Heidari | 2022 | ADIPOSE | Adipose + PRP | Hip | 1 | 6 ml | N | Y | Y | 12 | Y |
|  | Hernigou | 2021 | BMAC | BMAC (subchondral) | Knee | 1 | 20 ml | Y | N | Y | 12 | Y |
|  | Herold | 2017 | ADIPOSE |  | Hand/wrist | 1 | 1 ml | N | N | Y | 12 | Y |
|  | Holzbauer | 2022 | ADIPOSE |  | Hand/wrist | 1 | 1 ml | N | N | Y | 24 | Y |
|  | Hudetz | 2017 | ADIPOSE |  | Knee | 1 | 4-15 ml | N | U | N | 12 | Y |
|  | Hudetz | 2019 | ADIPOSE |  | Knee | 1 | 5 ml | N | U | N | 12 | Y |
|  | Hussein | 2021 | BMAC | Autologous conditioned serum, HA | Knee | 1 | 8 ml | N | U | Y | 12 | Y |
|  | Iacono | 2023 | ADIPOSE |  | Foot/ankle | 1 | 7 ml | N | Y | Y | 24 | Y |
|  | Jeyaraman | 2024 | BMAC | BMAC, Saline | Knee | 1 | 10-12 ml | N | N | Y | 12 | N |
|  | Jeyaraman | 2024 | BMAC |  | Knee | 1 | 10-12ml | Y | N | Y | 24 | Y |
|  | Kaszyński | 2022 | ADIPOSE | PRP, negative control | Knee | 1 | not reported | N | No | N | 12 | N |
|  | Kim | 2020 | ADIPOSE |  | Knee | 1 | 17 ml | N | N | Y | 12 | N |
|  | Kuebler | 2022 | BMAC |  | Knee | 1 | 10 ml | N | Y | N | 6 | Y |
|  | Louis | 2021 | ADIPOSE | Adipose + PRP | Knee | 1 | 10 cc | N | Y | Y | 6 | Y |
|  | Mautner | 2019 | BMAC | Adipose | Knee | 1 | 8 ml | N | Y | N | 12 | N |
|  | Mautner | 2023 | BMAC | SVF, umbilical cord tissue, corticosteroid | Knee |  | not reported | N | Y | N | 12 | Y |
|  | Meyer-Marcotty | 2022 | ADIPOSE |  | Hand/wrist | 1 | 0.5-1 ml | N | N | Y | 44 | Y |
|  | Miles | 2022 | ADIPOSE |  | Knee | 1 | 19.75 (3.5-36) ml | N | N | Y | 12 | Y |
|  | Muthu | 2024 | BMAC | BMAC (different doses) | Knee | 1 | not reported | N | U | Y | 12 | Y |
|  | Muthu | 2024 | BMAC | BMAC (different doses) | Knee | 1 | not reported | N | U | Y | 12 | Y |
|  | Muthu | 2024 | BMAC | BMAC (different doses) | Knee | 1 | not reported | N | Y | Y | 12 | Y |
|  | Natali | 2021 | ADIPOSE |  | Foot/ankle | 1 | 5 ml | N | Y | Y | 24 | Y |
|  | Natali | 2023 | ADIPOSE |  | Hip | 1 | 4 ml | N | Y | Y | 36 | Y |
|  | Natali | 2023 | ADIPOSE |  | Shoulder | 1 | 4 ml | N | Y | Y | 36 | Y |
|  | Pabinger | 2024 | BMAC |  | Knee | 1 | 5ml | N | U | Y | 60 | Y |
|  | Panchal | 2018 | ADIPOSE |  | Knee | 1 | not reported | N | Y | Y | 12 | Y |
|  | Pintore | 2023 | ADIPOSE | BMAC | Knee | 1 | 10 ml | N | N | Y | 6 | N |
|  | Rasovic | 2023 | BMAC |  | Knee | 1 | 10 ml | N | U | N | 12 | Y |
|  | Richter | 2024 | ADIPOSE | Corticosteroid, saline | Knee | 1 | 7 ml | N | N | N | 12 | Y |
|  | Rodriguez-Fontan | 2018 | BMAC |  | Knee; Hip | 1 | 12 ml | N | Y | Y | 24 | Y |
|  | Screpis | 2022 | ADIPOSE |  | Knee | 1 | 8mL | N | U | Y | 24 | Y |
|  | Shapiro | 2017 | BMAC | Saline | Knee | 1 | 15 ml | N | Y | Y | 12 | Y |
|  | Shapiro | 2019 | BMAC | Saline | Knee | 1 | 15 ml | N | Y | Y | 12 | Y |
|  | Shaw | 2018 | BMAC |  | Knee | 4 | 6 ml | N | Y | N | 3 | Y |
|  | Silvestre | 2023 | BMAC |  | Knee | 1 | 10-19 ml | N | Y | Y | 12 | Y |
|  | Smith | 2023 | BMAC |  | Knee | 1 | 8-10 ml | N | Y | Y | 6 | N |
|  | Themistocleous | 2018 | BMAC |  | Knee | 1 | 10 ml | Y | U | Y | 11 | Y |
|  | Tsitsilianos | 2022 | BMAC |  | Hip | 1 | 6-8 ml | N | Y | Y | 12 | N |
|  | Varady | 2020 | BMAC |  | Knee | 1 | 10 ml | Y | Y | Y | 3 | Y |
|  | Vinet-Jones | 2020 | ADIPOSE |  | Shoulder | 1 | 7 ml | N | Y | Y | 12 | N |
|  | Vitali | 2022 | BMAC |  | Knee | 1 | 7-10 ml | N | N | N | 6 | N |
|  | Wells | 2021 | BMAC |  | Knee | 1 | 10 ml | N | N | N | 12 | Y |
|  | Winter | 2023 | ADIPOSE | Adipose+ PRP, PRP, saline | Hand/wrist | 1 | 1.5 ml | N | N | Y | 24 | Y |
|  | Yu | 2023 | ADIPOSE |  | Knee | 1 | 6-8 ml | N | U | N | 18 | Y |
|  | Zaffagnini | 2022 | ADIPOSE | PRP | Knee | 1 | 5 ml | N | N | Y | 24 | Y |
|  | Zannoni | 2024 | ADIPOSE |  | Knee | 1 | not reported | N | Y | N | 12 | Y |

| **Abbreviation** | **Definition** |
| --- | --- |
| ADIPOSE | Autologous adipose tissue–derived therapy |
| BMAC | Bone marrow aspirate concentrate |
| HA | Hyaluronic acid |
| LP‑PRP | Leukocyte‑poor platelet‑rich plasma |
| MSCs | Mesenchymal stem cells |
| PL | Platelet lysate |
| PRP | Platelet‑rich plasma |
| SVF | Stromal vascular fraction |
| US | Ultrasound |
| Y | Yes |
| N | No |
| U | Unknown |
